# "Dental Legislation," Review of an Address by Dr. C. W. Barrett

**Published:** 1876-04

**Authors:** James B. Hodgkin


### ARTICLE II.

### "*Dental Legislation.*"—*Review of an Address by C. W. Barrett.*

Read before the New York State Dental Society.

BY J. B. HODGKIN, D. D. S.

Whether the mass of practicing dentists realize it or not, assuredly the day is fast approaching when some well-defined and legal status must be assigned our profession. The present anomalous condition, a necessary consequence perhaps of the rapid and irregular growth of dentistry, cannot much longer exist without serious damage to the prospects of the profession. That it must assume a higher position, that it must occupy more advanced ground, is a necessity growing out of our near relation to things about us which do stand high, and necessary also from the leaven which is at work within us,—thoughtful minds and earnest aspiring spirits,—and these are rapidly multiplying.

That dentistry is but a branch of medicine, must of course be apparent to all who for a moment reflect upon the subject. A minor and subordinate part it may be, but nevertheless an integral portion of that science ; and is—dispute it and sneer at it as medical men proper may—in its sphere and in its more cultivated aspect, quite as scientific. And as medicine is under the protection of the law, it is time that dentists were considering their legal relations, and the question of legal protection. It has been demonstrated we believe that they are liable to prosecution for malpractice, and that a tooth has a real and tangible value which can be estimated and sued for ; and it is worth while that the law should discriminate in favor of those who are, by education and training, in a position not to commit those crude and gros, errors which stamp the pretendor, and against the upstart and the charlatan.

That the profession can and should have this protection, and that it may have it, is the gist of an address lately delivered by Dr. W. C. Barrett, of Warsaw, New York, before the New York State Dental Society, and published in the Dental Register, and also in pamphlet form. The address details the necessity for, and the benefits of State Legislation in the interests of the dental profession, and urges *organization* as its first aim. We quote the opening paragraph:

"Whether, in the estimation of the world, Dentistry shall be classed as a liberal and learned profession, or shall degenerate into a mere handicraft trade, rests, in a great, measure, with the dentists of to-day. What shall be its future, is a question well worthy the attention of every practitioner, who has a thought above the mere bread and butter aspect of the case."

Introducing the subject thus, Dr. Barrett proceeds to consider the question as to dentistry being a medical specialty, and assumes that the pursuit of a specialty presupposes an acquaintance with general practice; and a subsequent divergence and devotion to a particular branch. He thinks that "the resentment shown by the medical pro fession proper, toward our rather impertinent claims is but the exhibition of a proper spirit on their part."

It is certainly true, that dentists have no right to expect full recognition as medical specialists until they bring forth fruits worthy of such recognition. So long as dentists run after physicians to administer anæsthetics for them in their (the dentists') offices; so long as maxillary fractures are the *bete noir* of the profession, and operations plainly belonging to facial surgery are relegated to the general surgeon—so long in a word as a general subserviency to the claims of the medical profession is seen every where among us, is it any wonder that the D. D. S. is recognised as "a badge of partial culture?"

Really the relations of dentistry to medicine must be discussed on hypothetical grounds, rather than on conditions that exist. If dentists expect that general recognition which will one day be accorded them, they must be something which they are not now. They must occupy higher and more advanced ground. It is not sufficient that a few advanced men here and there, scouts as it were, have penetrated into the territory so jealously guarded. The move must be a general one of rank and file. We must show not only that we have a title to the position, but that we can assume and maintain it. It is to stimulate such a move that Dr. Barrett writes:

"We possess all the elements of a profession, but so long as we are but an unorganized mass, with no responsible head, we cannot wield our true professional influence. We have schools, a literature, learned men, enthusiastic students in science, whose fame is not circumscribed by the boundaries of state or county: but a mob, even of officers, is not an army. For proper and effective organization, there must be some central heart, from which shall radiate, and toward which shall converge all the life currents, so necessary to the suppor tof an organism. Heretofore, the efforts for the advancement of Dentistry, have been individual and desultory. While each was anxious that his chosen profession should secure the respect of the other, there has not been a necessary harmony of action. All have desired recognition from our near kindred, medicine. But there has been no unit for their acknowledgement; there has been no responsible head, to which the avowal might be made. Themselves thoroughly organized in schools, with clearly defined lines of demarcation, they have hesitated to give the hand of fellowship to an irresponsible, disorganized aggregation, however respectable otherwise. Having a settled habitation, they are distrustful of the dwellers in tents."

Some barrier, marking the confines of our profession must be erected, that we may know and those outside may know what is its scope, aims, designs. Until this is done, until some distinction is made between the usurper and pretender, and the true and worthy practitioner, it is in vain to expect formal recognition and fraternity with those who for themselves possess these safe-guards and zealously guard them. This is well recognised as necessary in other professions, Law, Theology and Medicine. A diploma or its equivalent, is in their profession a condition precedent to practice.

Granting the medical diploma is rated at its true worth by those who are aware of its real value, or want of value, and who are posted as to the ease with which it is obtained; still, public sentiment has been educated to a firm belief in its importance, and of its being absolutely essential. And this feeling on the part of the uninitiated, the medical profession is ever on the alert to stimulate and foster. How, in those States where the non-graduate of medicine is so lucky as not to be absolutely prohibited by law from prescribing and taking his fee, he is professionally ostracised, is contumeliously thrust aside, is a matter of familiar knowledge. No amount of skill,—and it is certain that some of those who have "picked up" medicine are possessed of not a small amount of this; no amount of study or natural gifts, are allowed as an offset to the degrading admission the unfortunate is obliged to make,—"I have no diploma." Through this gate, and through this gate only must we pass in order that we may join the ranks of the favored. Only in this way can we be possessed of the immunities and privileges of the "regular" practitioner.

Until the dental profession shall establish some such system as this, and rigidly adhere to it; until they shall assume some such attitude as has been assumed by the medical profession, and shall show by their acts that they are sincere in assuming it, and that they will not fraternize with those who are reaping the benefits of their studies without sharings its toils, they must not expect better things than they now have. Dr. Barrett proceeds:

"If we would take the place which of right belongs to us, we must come up to the high plane occupied by the other professions. When, possessed of equal learning, we aspire to equal recognition, we must first take equally firm ground, and give security that we will not recede from that position. In but very few States have we an organized existence. There is no central point around which may crystallize the elements of a profession. We are generally an irresponsible jumble of learned and ignorant, capable and incapable, competent men and quacks, the latter greatly in preponderance, and this undisciplined mob has pressed forward and demanded a place beside the professions whose immunities have for centuries been jealously guarded from interlopers and charlatans. Instead of our exemplifying the true professional principle, and endeavoring to disseminate scientific truths and knowledge, it has until quite lately been the practice for dentists to jealously guard their operating rooms and laboratories from the prying inspection of their neighbors, and to hoard up the poor scraps of knowledge they had acquired, lest their professional brother might benefit thereby. Or more probably, the possessor of some fancied or real improvement straightway secured a patent upon it, and began to vend out his knowledge. Dental patents have done more to prevent our attaining self-respect and the respect of others, than any one thing. He is scarce worthy to be called a member of a liberal profession, who only studies the field of science that he may make it tributary to his own selfish gratification;—who has no love for original investigation for its own sake, but only pursues it because it is capable of being turned into a source of personal profit;—who would use Promethean fire, stolen from heaven, to cook his steak by, and turn Pegasus into a cart horse. This is not the conduct of him who is worthy to be called a member of a learned profession; whose study is that he may gain knowledge for the purpose of imparting it to his professional brother, that thus his beloved profession may, *as a whole*, be raised, and the scientific education of its members, as a class, be thereby perfected."

To establish an *esprit du corps*, and bring about a proper professional feeling, requires organization. In no other way can it be done. The labor of a solitary thinker or worker here and there in the matter can avail little, so long as there is no concert of action. The field of dentistry most certainly belongs to dentists. They have won it fairly, by hard work. It is ground entirely unexplored by the medical profession, and they are slow even now to recognise the value of the fund of knowledge which the labor of dentists has brought them. If they avail themselves of the light which dentistry has thrown on obscure pathology, they are in some sort incredulous as to its source. Thoroughly explored and cultivated as this field has been by the dentist, it is due the profession that they should legally possess it. As Dr. Barrett says, " having subdued a certain portion of the field of science and adopted it as our heritage, having thoroughly explored and cultivated it, some legal recognition is necessary that our title may be made good."

How shall dentistry free itself from the gross elements which so trammel its progress onward, which are so sadly in the way of its advance? Not by prohibition. Dental Legislation, which means this must fail of its aims. It is important that the large mass of those outside of the colleges should be conciliated, for they vastly outnumber the graduates. According to Dr. Morgan, of Nashville, Tennessee, not eight per cent. of all the dentists practicing in the South are graduates of any college; and out of two hundred and fifty practicing dentists in Tennessee, says the same au thority, not twenty hold dental diplomas. It is probable that the proportion is somewhat greater in other places, than the localities named, but not greatly. So that, while it is important to institute some means to protect ourselves and benefit the people by delivering them from the hands of the pretentious and ignorant, it is well to see that these means are such as not to array in hostility against us this immense outside majority.

" In the consideration of Dental Legislation, men are apt at once to jump to the conclusion that it means prohibition, and this it is which has arrayed against it all the quacks, and many of the qualified practitioners. A greater mistake could not be made. If interdiction of any class ever be allowable, it comes only as a subsequent. The first aim of all dental legislation should be ORGANIZATION. We ought not to commence with enactments against any particular class, till they have had time to bring forth works meet for repentance. We should strive to convince the ignorant and incapable that we are laboring for the benefit of all, and thus secure their good will, and not render ourselves liable to the charge of proscription. To this end, while not throwing down one bar to their admission into our societies, we should smooth the way to the obtaining a proper professional education."

This will take a long time. It is much to be feared, looking at the matter from a practical point of view, that most of those who are now practicing will be ready to retire or will be retired by the hand of death or disability, before we see this field in good order. Considering the slow growth of medical science, it is comforting to think that we have done as well as we have, and really we may pride ourselves on deserving a great deal of credit. Still, as Dr. Barrett says, "when the other professions see a responsible head to a body of learned men, who are devoted to the study of science in their own particular field, recognition and acknowledgement will not long be delayed." But want of organization is fatal to the first step—indeed organization must precede any other step in this direction.

Three kinds of legislation have been procured in the interests of dentistry so far in this country. In Ohio, New Jersey and Georgia, stringent laws have been enacted, prohibiting non-graduates from practicing. But little attempt however is made to enforce these laws, and where the attempt has been made, it has met with much opposition. The following statistics from Ohio will show what legislation has done, and also the relative proportion of the two classes, graduates and non-graduates, to each other.

"In the year 1873, the whole number of dentists practicing in Ohio was 433. Of this number, 99 or about 23 per cent were graduates. Of the remainder, 142, or about 43 per cent. had passed the examining board, while 192, or about 44 per cent. of the whole had done neither. Of all the dentists of Ohio, 56 per cent. were legally qualified to practice while the remainder, nearly half, were living in open violation of the law."

Incorporation of Dental Societies is another means by which dentists have sought to protect themselves. No obvious good has seemed to result from this so far.

The third kind of legislation is represented by the New York State enactment, which gave rise to the Dental Society of the State of New York.

"The object of the orginators of this law, was to thoroughly organize the profession of the State, establish for it some definite limits, secure perfect legal recognition, and institute a criterion by which the good might be distinguished from the bad, thus throwing the whole moral weight of the community for the one and against the other. So well has it succeeded in its work, especially in the country districts, that I propose to here give a review of its provisions, setting it up as a kind of example of this class of legislation.

The New York State Dental Law divides the state for dental purposes into eight districts, following the boundaries of the judicial divisions as establishing lines familiarly known, and districting the State into convenient and proper provinces for the purpose sought. In each district the law establishes a dental society, and eight delegates from each district dental society, elected to serve for the term of four years, together with an indefinite number of permanent members, of whom five may be elected each year, to be selected from among the eminent practitioners of the State, and two representatives from each dental college in the State, makes up the society, which the law provides shall meet at least once a year in the Capitol at Albany. Its expenses are provided for by the levying of dues upon each district society, by the dues of the permanent members, and by the fees to be collected upon conferring its diploma. District societies are subordinate to this State society, and must make to it an annual report of their status and proceedings. Each district society elects a board of censors, before whom must appear every student in dentistry who proposes to commence its practice. Should he successfully pass the examination of the board, he becomes entitled to the diploma of the society and to membership therein, and is permitted to open an office; but without such diploma it is made unlawful for any one to commence the practice of dentistry, *subsequent to the passage of the law.*"

There is no penalty for such as fail to conform to this law, save the opprobium of being constructively branded as quacks. The "board of censors" meets annually or oftener if necessary, examines applicants, and such as pass are certified to the State society, which can confer on the applicant the diploma and degree of "Master of Dental Surgery," (M. D. S.) The recipient of this diploma is required to sign a bond to "walk worthy of his vocation."

"There was no difficulty in obtaining the passage of the enactment, for there was nothing in it to arouse opposition in the legislature, and to-day the law and society are so strongly entrenched in the good will of the people, that further legislation might, if thought desirable, be easily procured. There has been no attempt at bluster; all has been done quietly but thoroughly, and while no astonishing revolution has overturned the existing order of things, the good effects of the law have been felt everywhere, and its originators have every year seen greater and still greater reason to congratulate themselves upon the original moderateness of their demands. If in the future it be thought advisable to press more stringent measures, the society having gained a firm foothold and having practically enforced previous legislation, will find it comparatively easy to obtain enactments establishing proper penalties for the infringement of the existing status."

This legislation, it is claimed, has wrought a great amount of good. It secures perfect organization, bands members of the profession in adjacent sections together, close professional intimacies are formed, and the profession throughout the State feel that they have a common interest in keeping up the society.

As to the establishment of the degree of M. D. S. (Master of Dental Surgery,) it is of course understood that it in no way conflicts with, or is a substitute for a degree from regular school, nor is it given as evidence of special scholastic training. It was instituted for the beneflt of a large class who, of necessity perhaps, had obtained their dental training at home; some, doubtless many, of whom are quite as well qualified to practice as those who have passed the ordeal of the schools. The degree simply determines the status of such; and if, as is stated, a strict examination is given them, they are respected as having complied with the terms of the law. This degree is quite largely sought after by regular graduates.

"It does not in any way relieve the student from the necessity of attendance upon a dental college, as the degree of Doctor of Dental Surgery only, indicates the possession of the necessary qualifications for the practice of dentistry while this is proof that he is putting to a reputable use his dental education, and is in successful practice. This degree, analogous to the Licentiate of Dental Surgery of Canada, though requiring much higher qualifications in the candidate for its possession, has been a great incentive to study on the part of the younger members of the profession, and indeed many of the older graduates have found it necessary to brush up considerably anticipatory to making their appearance before the Board of Censors. Instances have been where the candidate has been repeatedly rejected, only to go through another year's careful study and another trial."

Dental legislation, if even of this imperfect sort, must, it is not difficult to see, work much good,—must result in protection to the regular graduate. A few of its benefits are detailed by Dr. Barrett:

"It expressly entitles us to 'all the privileges and immunities heretofore accorded to the medical profession:' Under it, in the cities, dentists claim exemption from jury duty, and professional fees when summoned as professional witnesses. The annual reports of the society are made to the Legislature and are printed and published as official documents, the same as are the reports of the State medical societies, and the various charitable institutions under the patronage of the State. But the greatest advantage gained through legal enactment, is in the recognition of the claim of dentistry to an honorable position by the side of medicine, law and theology."

Dr. Barrett concludes with the hope that the other States will adopt some plan the equivalent of this, and that we may soon look forward to a National Dental Association.

There is one thing that must sadly hinder all attempts at improvement by union of forces. The petty jealousies, the bickerings, the enviousness and heartburnings which so mar our intercourse with each other, and which are seen so plainly to affect our association with each other, will baffle any attempts at organization, or so hinder it as to make it nugatory and useless. Until our dentists can realize that the interest of one is the interest of all, until they fully understand that the advancement of their profession as a science is better than the advancement of their individual practice, it is not probable that anything substantial or noteworthy in the way of organization will be accomplished. So long as dentists show by their acts that they do not desire professional fraternity, it is difficult to see the way to remedy existing evils. Concert of feeling must precede concert of action. How to get this is a perplexing problem.

